# Cardiac rehabilitation of Baduanjin exercise in coronary heart disease after PCI: A protocol for systematic review and meta-analysis of randomized controlled trials

**DOI:** 10.1097/MD.0000000000025501

**Published:** 2021-04-16

**Authors:** Xing-Xing Li, Zong-Jing Fan, Jie Cui, Quan Lin, Rui Zhuang, Rong-Peng Liu, Yang Wu

**Affiliations:** a Department of Graduate School, Beijing University of Chinese Medicine; b Department of Cardiology, Dongfang Hospital, Beijing University of Chinese Medicine; c Xiyuan Hospital, China Academy of Chinese Medical Sciences, Beijing, China.

**Keywords:** Baduanjin exercise, cardiac rehabilitation, coronary heart disease, percutaneous coronary intervention, protocol, randomized controlled trials, systematic review

## Abstract

**Background:**

Percutaneous coronary intervention (PCI) is an effective revascularization strategy in patients with coronary heart disease (CHD). However, recent studies had indicated that postPCI patients usually suffer from a low-quality life. Cardiac rehabilitation (CR) has been recommended by numerous guidelines in the clinic for these patients. And Baduanjin exercise can significantly benefit patients with CHD. Regrettably, the effect of Baduanjin exercise on postPCI patients is still not clear. Therefore, this systematic review and meta-analysis protocol is planned to explore the effect of Baduanjin exercise in patients with CHD who have undergone PCI.

**Methods:**

PubMed, Excerpta Medica Database, Cochrane Library, Web of Science, Wanfang Database, SINOMED, China Science and Technology Journal Database, and China National Knowledge Infrastructure will be searched for appropriate articles from respective inceptions until December 1th, 2020. Two reviewers will independently conduct article selection, data collection, and risk of bias evaluation. Disagreements will be resolved first by discussion and then by consulting a third author for arbitration. The primary outcome will include left ventricular ejection fraction. And the change in the scores on the Seattle Angina Questionnaire, SF-36 health survey scale, Zung Self-rating Anxiety scale and self-rating depression scale will be used as the secondary outcomes. RevMan 5.3 will be used for meta-analysis.

**Results:**

This systematic review and meta-analysis will explore whether Baduanjin exercise is an effective intervention in postPCI patients.

**Conclusion:**

This systematic review and meta-analysis will provide convincing evidence of Baduanjin exercise that specifically focuses on CR of Baduanjin exercise on CHD after PCI.

**Registration number:**

INPLASY202130065.

## 1 Introduction

Coronary heart disease (CHD), also known as coronary artery disease, is the most common cause of death worldwide.^[1]^ Percutaneous coronary intervention (PCI) is a common therapeutic strategy that can improve the survival rate of patients with CHD.^[2]^ During follow-up, however, there are still recurrences of cardiovascular events after PCI and patients usually suffer from a low-quality life.^[3]^ Cardiac rehabilitation (CR) has been recommended by numerous guidelines in the clinic for patients with CHD after PCI.^[4]^ Combined with routine therapy, exercise-based CR is a convenient and safe option, according to the guidelines from the American Heart Association.^[5]^ What's more, mild to moderate intensity physical exercise is reported to have protective effects on survival for patients postPCI surgery.^[6]^

As one of the traditional Chinese exercise, Baduanjin (also called Eight-Section Brocades) combines meditation with gentle movements and has been widely practiced in China to prevent and treat diseases.^[7,8]^ Researches showed that Baduanjin exercise as a complementary and alternative therapy for cardiovascular patients can improve the clinical outcome of cardiovascular patients and reduce the occurrence of adverse cardiovascular events.^[9]^ This exercise can activate a sequence of natural self-regulatory/self-healing mechanisms to stimulate the balanced release of endogenous neurohormones, and is easy to master in a short time and suitable for all age groups.^[10]^ Currently, growing numbers of randomized controlled trials (RCTs) were conducted to explore the effect of Baduanjin exercise on the cardiac rehabilitation of patients with CHD after PCI. Regrettably, the sample sizes of these studies are small.

Therefore, we will perform a systematic review of RCTs to evaluate the effects of Baduanjin exercise on cardiac rehabilitation in patients with CHD after PCI, so as to provide more powerful evidence for treating postPCI CHD patients in clinic.

## 2 Methods

### 2.1 Study registration

This protocol has been registered in INPLASY (INPLASY202130065). In addition, it also has been prepared based on the Preferred Reporting Items for Systematic Reviews and Meta-Analyses Protocols (PRISMA-P) statement guidelines.^[11]^

### 2.2 Eligibility criteria

#### 2.2.1 Study type

We will collect all available RCTs of Baduanjin exercise-related therapies for CHD after PCI, regardless of blinding, publication status, or region, but the language is limited to Chinese and English.

#### 2.2.2 Participants

Patients at least 18 years’ old with CHD who received Baduanjin exercise intervention after PCI will be included without limitation of race, sex, education, and economic level.

#### 2.2.3 Interventions

Using Baduanjin exercise for CHD patients postPCI surgery. Included variation in intensity, frequency and duration will be accepted.

#### 2.2.4 Outcomes

The primary outcome will included left ventricular ejection fraction. The secondary outcomes will included Seattle Angina Questionnaire, SF-36 health survey scale, Zung Self-rating Anxiety scale, and self-rating depression scale.

### 2.3 Exclusion criteria

The specific criteria are as follows:

1. non-RCTs, case reports, animal trials, research advances, reviews, expert experience and conference articles;
2. patients who received other exercise measures in addition to Baduanjin exercise;
3. duplicate articles, studies with the data were incorrect, inconsistency or incomplete and unavailable literature.

### 2.4 Search strategy

We will search 4 English electronic databases and 3 Chinese literature databases for studies published before November 29th, 2020: PubMed, Excerpta Medica Database, Cochrane Library, Web of Science, Wanfang Database, SINOMED, China Science and Technology Journal Database, and China National Knowledge Infrastructure. And there is no restriction for language. Take PubMed as an example, and details of search strategy are shown in Table 1. Moreover, manual retrieval will be performed on baidu academic, Google academic, books, impurities, and conference materials in order to obtain all the materials related to this study as comprehensively as possible. This work will be completed by 2 independent reviewers and, in cases where they have a disagreement, a third person will be asked for advice.

**Table 1:** Search strategy in PubMed database.

Number	Search terms	Number	Search terms
#1	Baduanjin exercise	#14	PCI
#2	Baduanjin	#15	Coronary Intervention, Percutaneous
#3	Eight section brocades	#16	Revascularization, Percutaneous Coronary
#4	#1OR#2OR#3	#17	Coronary Revascularization, Percutaneous
#5	Coronary heart diseases	#18	#13OR#14OR#15OR#16 OR#17
#6	CHD	#19	Randomized controlled trial
#7	Coronary artery disease	#20	Randomized trial
#8	Angina pectoris	#21	Clinical trial
#9	Myocardial infarction	#22	Clinical study
#10	Acute coronary syndrome	#23	Controlled study
#11	Cardi^∗^	#24	#19OR#20OR#21OR#22OR#23
#12	#5OR#6OR#7OR#8OR#9OR#10OR#11	#25	#4 AND#12 AND#18 AND#24
#13	Percutaneous coronary intervention		

### 2.5 Data screening and extraction

**Figure 1:**
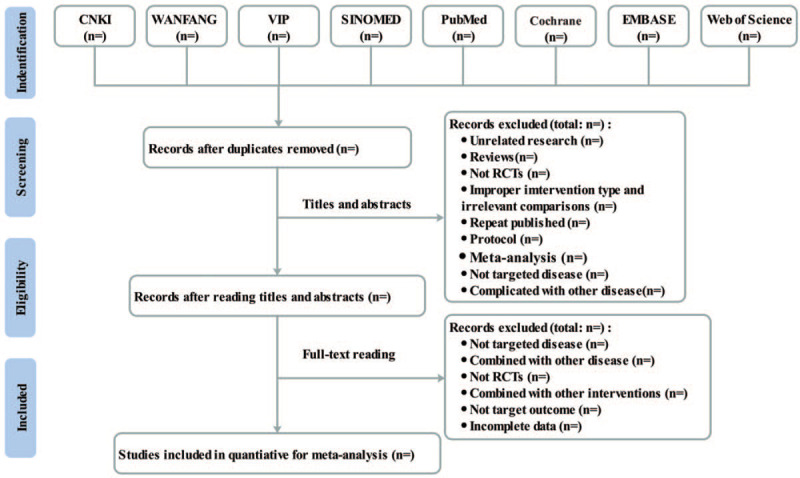
Flow diagram for study selection.

All authors will receive specific training at PRISMA-P before the implementation of the study. Review process will be independently conducted by 2 authors, using blinding to reduce bias. Disagreements will be resolved first by discussion and then by consulting a third author for arbitration. Endnote X7 will be used to manage and screen literature. RCTs that are duplicative and do not meet the eligibility criteria will be removed. Details of literature screening process is shown in Figure 1.

Then, Excel 2011 software will be used to extract relevant data. The relevant information that needs to be extracted is as follows:

1. basic information: title, the first author's name, year of publication;
2. study characteristics: interventions, course of treatment, duration;
3. subjects characteristics: sample size, gender, age, and underlying disease;
4. outcomes and specific adverse reactions.

When there is uncertain information in included studies, the final decision will be made through discussion among team members.

### 2.6 Risk of bias and quality assessment

Two colleagues will separately assess the quality of include RCTs via the tool of Cochrane Collaboration, and in the case of disagreement, a third author will be asked for advice. The review items comprised: random sequence generation, allocation concealment, blinding, incomplete outcome data, selective reporting and other bias. According to the characteristics of the included literature, the reviewers can classify it as low, high, or unclear risk of bias.

### 2.7 Statistical analysis

All data will be statistically analyzed using RevMan5.3 software from the Cochrane Collaboration. Discontinuous variables will be expressed as the risk ratio with its 95% confidence interval (CI). For continuous data, if the unit or the measurement instrument is consistent, the mean difference with 95% CI will be used, and if not, the standard mean difference with 95% CI will be selected. The χ^2^ test and *I*^2^ test will be used to assess the heterogeneity among the included RCTs.^[12]^ Fixed-effect model will be utilized when heterogeneity is low (*P* ≥ .05, *I*^2^ ≤ 50%). But when high heterogeneity occurs (*P* < .05, *I*^2^ > 50%), we will further analyze its potential sources from 3 aspects: clinical heterogeneity, methodological heterogeneity, and statistical heterogeneity.

We will evaluate clinical heterogeneity first: if there is evident clinical heterogeneity, subgroup analysis will be performed; if clinical heterogeneity is evident and subgroup analysis cannot be conducted, descriptive analysis is only used. After excluding clinical and methodological heterogeneity, statistical heterogeneity should be considered and a random-effect model was used.^[13]^

#### 2.7.1 Subgroup analysis

If necessary, subgroup analysis will be performed to reduce the clinical heterogeneity between groups according to differences in the types of CHD, treatment period, and frequency, and so on.

#### 2.7.2 Sensitivity analysis

We will carry out sensitivity analyses in order to evaluate reliable results. The methods include changing the type of analysis methods (random-effects model or fixed-effect model), eliminating each of the included studies one by one and then combine the effect quantities.

#### 2.7.3 Dealing with missing data

If necessary, original authors will be contacted where possible to obtain missing information. Studies that cannot be supplemented or corrected with the required information will be eliminated.

#### 2.7.4 Assessment of reporting biases

A funnel chart will be conducted to evaluate the publication bias if there are no less than 10 studies included.^[13]^

### 2.8 Ethics

The patients’ privacy is not involved in the study, so ethical approval is not needed.

## 3 Discussion

CR refers to the coordinated sum of the interventions needed to ensure the optimal physical, psychological and social conditions of patients with chronic or acute cardiovascular diseases so that they can preserve or resume optimal social functioning through their own efforts and slow or reverse disease progression by improving healthy behaviors.^[14]^ This comprehensive intervention may involve multiple therapies, such as exercise, risk factor education, behavior change, psychological support, and strategies for traditional risk factors for cardiovascular disease. Studies have shown that exercise training has direct benefits on the heart and coronary vasculature.^[15]^

Baduanjin exercise arise from eastern traditional culture and has been developed and matured under the background of traditional philosophy, the theory of Yin and Yang, the theory of the 5 elements, the theory of meridians and other traditional Chinese theories.^[16]^ As a common kind of low-intensity aerobic exercise, Baduanjin exercise can significantly benefit patients with cardiovascular diseases and reduce the occurrence of cardiovascular risk factors.^[17]^

In recent years, Baduanjin exercise has become increasingly popular and accessible in Western countries. However, the evidence of Baduanjin exercise for postPCI CHD patients lacks comprehensive system evaluation. In the present study, we will review all relevant clinical studies at home and abroad to evaluate the CR of Baduanjin exercise in CHD after PCI and to provide powerful evidence for potential treatment of Baduanjin for CHD after PCI in future utility. We therefore expect, and hope, that the current study can provide valuable information to patients, physicians, and health authorities.

## Author contributions

**Conceptualization:** Xing-Xing Li, Yang Wu.

**Funding acquisition:** Yang Wu.

**Methodology:** Quan Lin, Rui Zhuang, Rong-Peng Liu.

**Project administration:** Zong-Jing Fan, Jie Cui.

**Writing – original draft:** Xing-Xing Li.

**Writing – review & editing:** Xing-Xing Li, Yang Wu.
